# A global internal tide modeling framework for improving satellite observations of fine-scale ocean circulation

**DOI:** 10.1126/sciadv.aee1885

**Published:** 2026-06-03

**Authors:** Badarvada Yadidya, Brian K. Arbic, Edward D. Zaron, Jay F. Shriver, Maarten C. Buijsman, Eric P. Chassignet, Loren Carrère, Michel Tchilibou

**Affiliations:** ^1^ Department of Earth and Environmental Sciences, University of Michigan, Ann Arbor, MI, USA.; ^2^ Center for Ocean-Atmospheric Prediction Studies, Florida State University, Tallahassee, FL, USA.; ^3^ Research School of Earth Sciences, Australian National University, Canberra, ACT, Australia.; ^4^ College of Earth, Ocean, and Atmospheric Sciences, Oregon State University, Corvallis, OR, USA.; ^5^ Naval Research Laboratory, Stennis Space Center, MS, USA.; ^6^ Division of Marine Science, University of Southern Mississippi, Stennis Space Center, MS, USA.; ^7^ Collecte Localisation Satellites, 31520 Ramonville-Saint-Agne, France.

## Abstract

Small-scale oceanic eddies and filaments mediate the vertical exchange of heat and carbon within the global ocean. The Surface Water and Ocean Topography (SWOT) mission resolves these features through wide-swath interferometry, but internal tides often mask these observations. Non–phase-locked internal tides present special difficulty because they vary with the evolving ocean background. We show that this chaotic variability can be predicted. We use a data-assimilative ocean forecast model to resolve the mesoscale environment and separate tidal signals from the broader circulation. The model captures the organized structure of these incoherent waves in the independent SWOT measurements. Correcting for the total (phase-locked plus non–phase-locked) internal tide signal reduces the error by 59% relative to current empirical methods. These findings show that non–phase-locked internal tides become predictable when the evolving ocean state is explicitly modeled. Our framework allows wide-swath altimetry to more accurately map climatically important fine-scale dynamics in the ocean.

## Teaser

Advanced tidal modeling helps wide-swath satellite missions resolve the small-scale eddies that drive ocean heat transport.

## INTRODUCTION

A turbulent circulation of eddies and filaments governs the ocean’s ability to absorb heat and carbon (*1*, *2*). The internal tide obstructs efforts to observe these climate-critical features from space. Generated by the interaction of barotropic tidal flows with undersea topography (*3*, *4*), these kilometer-scale internal waves obscure satellite measurements because their signals often mimic the very circulation scientists need to measure. Internal tides also operate as a cornerstone of the global climate system in their own right. They propagate for thousands of kilometers (*5*, *6*), drive a notable fraction of the diapycnal mixing that maintains ocean stratification (*7*, *8*), and cascade energy down to small-scale turbulence (*9*, *10*). It is important to map internal tides for their own sake and to make more accurate maps of small-scale eddies.

For more than three decades, conventional nadir-altimetry satellites provided one-dimensional traces of the sea surface, restricting our view of its two-dimensional structure (*11*, *12*). The recently launched Surface Water and Ocean Topography (SWOT) mission fundamentally changes this paradigm (*1*, *13*, *14*). Using wide-swath radar interferometry, SWOT delivers the first high-resolution, two-dimensional maps of sea surface height (SSH), resolving ocean features down to scales of 5 km (*15*, *16*).

This expanded capability, however, exposes a fundamental observational barrier. SWOT’s high-resolution measurements capture the energetic and complex internal tide field at unprecedented scales, complicating the differentiation of climate-regulating, eddy-driven transport from transient tidal energy. A further challenge stems from the internal tide’s two-component nature. The first component, which is phase locked (or coherent) with the astronomical tides, is predictable and has been robustly mapped (*6*, *17*–*21*). The second, a non–phase-locked (or incoherent) component, has remained a critical blind spot. Time-evolving mesoscale eddies, wave-wave interactions, and stratification constantly modulate this highly variable component (*22*–*24*).

The non–phase-locked tide creates a fundamental ambiguity because its spatial scales and amplitudes closely match the mesoscale features SWOT targets. The empirical correction atlases developed in the pre-SWOT era therefore fail to meet this emerging challenge, as they only account for the predictable, phase-locked component (*19*, *20*, *25*, *26*).

We resolve this impasse by demonstrating that the chaotic non–phase-locked tides are substantially predictable. Our approach is based on an ocean forecast model that accurately captures the evolving mesoscale dynamics and background stratification and thus inherently simulates the resulting non–phase-locked signals (*27*). Modern ocean forecasting systems achieve this by integrating physical principles, high-resolution bathymetry, and atmospheric forcing with the assimilation of nadir altimetry and in situ streams, including Argo profiles and drifters (*28*). By constraining the four-dimensional mesoscale environment, these systems accurately position eddies and represent the three-dimensional stratification essential for realistic propagation of internal tides and their modulation by the evolving background.

We test this hypothesis using an 18-month global simulation of a data-assimilative forecast model [the HYbrid Coordinate Ocean Model (HYCOM) (*29*, *30*)]. The model explicitly captures internal tide generation and propagation via concurrent atmospheric and astronomical tidal forcing. To prevent aliasing of diurnal and semidiurnal cycles, we archived outputs at hourly intervals (see Materials and Methods for more details). Although the model assimilates nadir altimeters and in situ observations, we intentionally excluded SWOT data from the assimilation process to ensure an independent validation. We show that this physics-based simulation accurately reproduces not only the phase-locked internal tides but also a large fraction of the organized, fine-scale structure of the non–phase-locked variability. This approach offers a refined pathway for correcting satellite data and maximizes the utility of high-resolution altimetry.

## RESULTS

### Evaluating the predictable internal tide

**Fig. 1.**
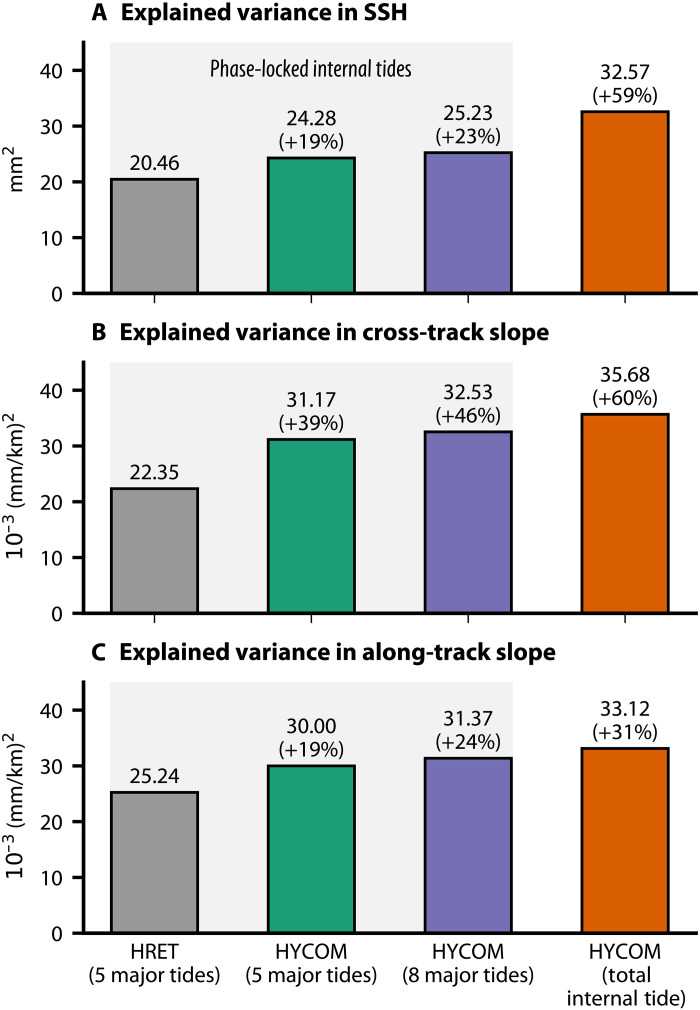
Explained variance of internal tide signals in SWOT observations across model configurations. Global mean explained variance for (**A**) SSH, (**B**) cross-track sea surface slope, and (**C**) along-track sea surface slope. The bar charts compare SWOT observations against four internal tide estimations: the empirical HRET model and three progressive products from the HYCOM simulation. The baseline comparison isolates the five principal phase-locked constituents (M_2_, S_2_, N_2_, K_1_, and O_1_) in both HRET and HYCOM. The third bar incorporates three additional weaker phase-locked constituents (K_2_, P_1_, and Q_1_) resolved by HYCOM. The final bar uses the total internal tide signal, comprising the eight-constituent phase-locked signal plus the non–phase-locked component. Numerical values indicate the absolute explained variance globally averaged across 584 SWOT passes, with the relative percentage increase over the HRET baseline in parentheses.

Before resolving the non–phase-locked internal tide, we evaluate the performance of the model’s phase-locked component, derived by harmonic analysis of the filtered SSH (see Materials and Methods). Our data-assimilative forecast model demonstrates strong agreement with observations. Globally, the model explains 19% more SSH variance in SWOT than the standard high-resolution empirical tide (HRET) atlas for the five principal constituents (Fig. 1A). Including weaker tidal constituents (e.g., K_2_, P_1_, and Q_1_), which empirical atlases such as HRET struggle to resolve from satellite data, increases this improvement to 23%.

The model yields even greater improvements in sea surface slope, the fine-scale gradient critical for calculating geostrophic currents and high-resolution bathymetry (*31*, *32*). Our framework explains 39% more cross-track slope variance and 19% more along-track slope variance than HRET for the five major tides (Fig. 1, B and C). Including the weaker tides increases these improvements to 46 and 24%, respectively, offering enhanced resolution for applications in physical oceanography (*15*) and marine geology (*32*).

**Fig. 2.**
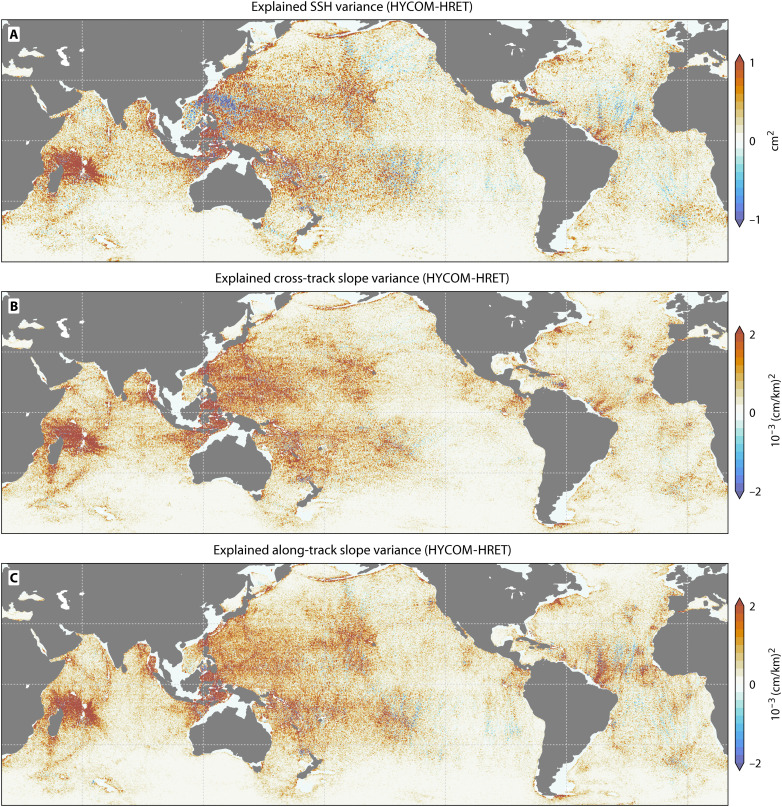
HYCOM explains more phase-locked internal tide variance in SWOT SSH and slope than HRET. (**A**) Difference in SSH variance in SWOT explained by HYCOM versus HRET empirical model based on five main tidal constituents contained in HRET (0.2° regridded bins). In red-shaded regions, HYCOM explains more variance than HRET, while HRET removes more variance in blue regions. (**B**) As in (A), but for the variance of the cross-track sea surface slope. (**C**) As in (A), but for the along-track sea surface slope.

These variance reductions occur globally rather than being restricted to specific regions. The model shows widespread improvements across all major ocean basins (Fig. 2) and captures particularly high variance over known internal tide generation hotspots such as the Hawaiian Ridge, the Luzon Strait, and the Mascarene Plateau. This spatial correspondence confirms that the model accurately represents internal tide generation dynamics.

**Fig. 3.**
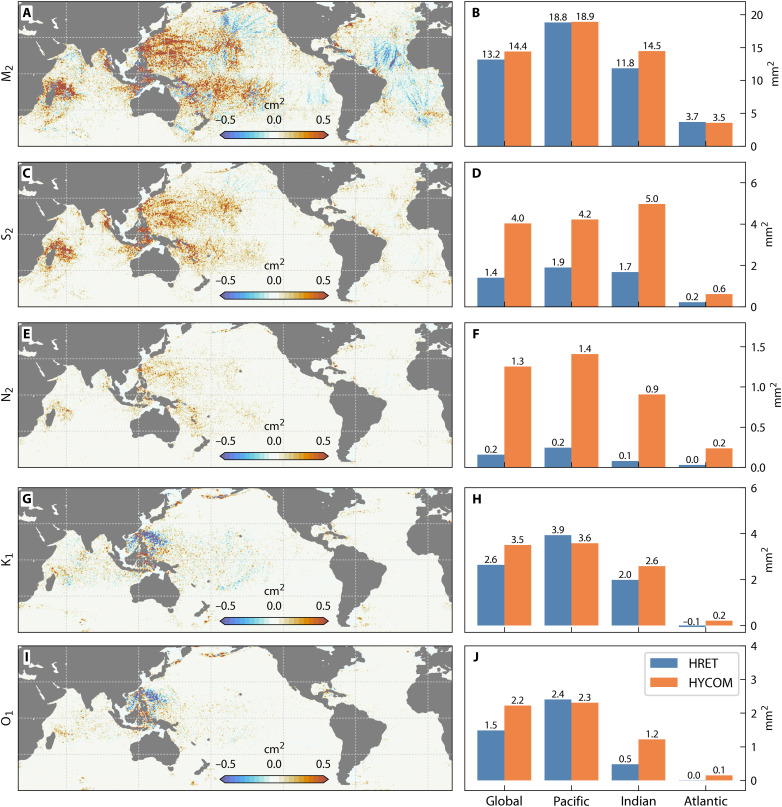
Difference between HYCOM and HRET in explaining phase-locked internal tide variance in SWOT SSH for major tides. (**A**, **C**, **E**, **G**, and **I**) Individual tidal constituents (M_2_, S_2_, N_2_, K_1_, and O_1_) showing frequency-dependent model skill (0.4° regridded bins). (**B**, **D**, **F**, **H**, and **J**) Adjacent bar plots summarize the mean explained variance for each ocean basin and for the aggregate signal, comparing HYCOM and HRET performance.

A constituent-by-constituent analysis identifies the specific drivers of this variance reduction (Fig. 3). The model accurately simulates the semidiurnal tides (M_2_, S_2_, and N_2_), where it consistently outperforms HRET. While HRET explains more M_2_ variance in certain localized regions, HYCOM’s performance remains superior on a global scale. Performance varies more spatially for the diurnal tides. For both K_1_ and O_1_ constituents, the empirical HRET model explains more variance than HYCOM across large areas of the Pacific. This suggests that regional inaccuracies in bathymetry and stratification, factors critical for diurnal tide generation, limit the current simulation’s accuracy in these areas.

We also acknowledge that HRET’s three-decade record averages over substantial interannual climate variability (e.g., El Niño–Southern Oscillation and Indian Ocean Dipole) that can modulate internal tides (*33*–*35*). In contrast, HYCOM’s 18-month record, while sufficient for dominant tides, is more susceptible to aliasing non–phase-locked energy into its phase-locked component. Despite this limitation, the model’s ability to resolve a richer vertical modal structure (*36*, *37*) yields substantial variance reductions. These results demonstrate that data-assimilative models provide a robust complementary tool to empirical analysis for mapping the complex, phase-locked internal tide field.

### Characterizing the non–phase-locked internal tide

**Fig. 4.**
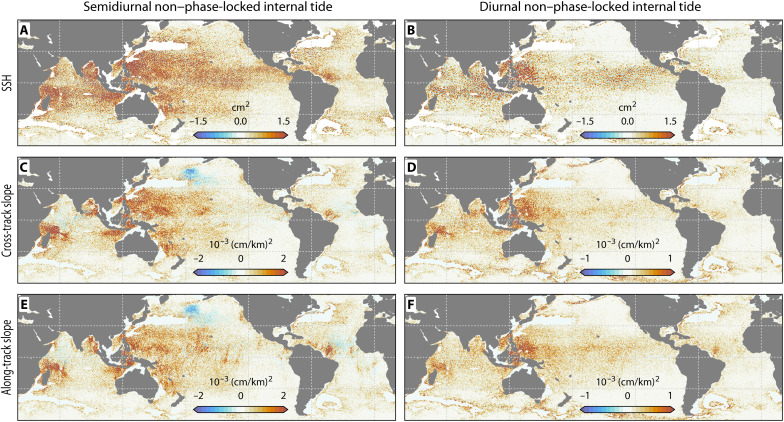
Assessment of HYCOM’s skill in representing non–phase-locked internal tides. The panels display the explained variance of incoherent internal tides in (**A** and **B**) SSH, (**C** and **D**) cross-track slope, and (**E** and **F**) along-track slope of SWOT. Each pair corresponds to the (A, C, and E) semidiurnal and (B, D, and F) diurnal bands, respectively. Red indicates high predictive skill; blue denotes negative variance explained. The analysis masks regions dominated by strong mesoscale eddy contamination (all maps are regridded onto 0.2° bins).

This dynamic capability becomes even more pronounced when characterizing the non–phase-locked internal tide. Our results demonstrate that this transient field constitutes a predictable and spatially organized component of the global SSH (Fig. 4). This predictability arises from the model’s capacity to physically simulate the evolving ocean state at a high (hourly) temporal resolution. Unlike traditional empirical models that rely on static harmonic averaging over long records, this prognostic modeling approach directly computes the dynamic interaction between the propagating tide field and the shifting mesoscale environment, capturing the rapid spatial modulations that occur on 10- to 30-day decorrelation timescales (*38*, *39*).

HYCOM explains a substantial fraction of this purportedly “unpredictable” variability. Globally, this amounts to 10.32 mm^2^ of total non–phase-locked explained variance (7.12-mm^2^ semidiurnal and 2.98-mm^2^ diurnal). Because the phase-locked and non–phase-locked signals are not strictly orthogonal over the finite SWOT sampling period, their variances do not sum linearly; thus, the total explained variance (10.32 mm^2^) exceeds the arithmetic difference between the total and phase-locked components shown in Fig. 1A. Furthermore, the explained variance slightly exceeds the model’s own predicted non–phase-locked variance (9.11 mm^2^). Mathematically, this ratio suggests a slight amplitude bias where the true non–phase-locked signal in the ocean is proportionally larger than the model’s prediction. This indicates the simulation is somewhat overdamped compared to the real ocean, resulting in an underestimated wave amplitude despite an accurate structural prediction. HYCOM exhibits the highest predictive skill near major topographic features (Fig. 4), capturing large-scale propagation pathways radiating from hotspots such as the Luzon Strait, the Indonesian archipelago, and Madagascar.

**Fig. 5.**
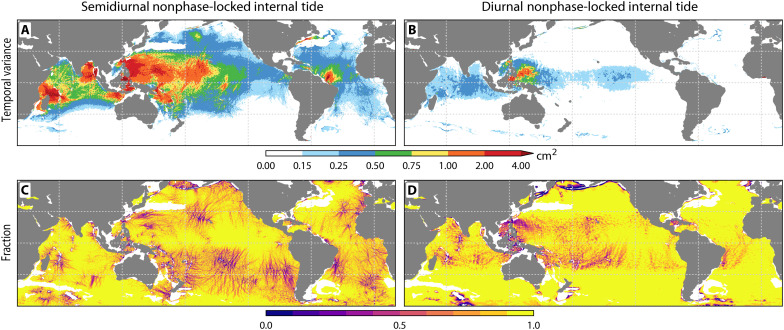
Global distribution of non–phase-locked internal tide variability. (**A** and **B**) Absolute variance of the non–phase-locked internal tide signal and (**C** and **D**) the fraction of total variance contributed by this incoherent component. The maps correspond to the (A and C) semidiurnal and (B and D) diurnal frequency bands, respectively. Energetic hotspots in the variance maps concentrate near major generation topography. In the fraction maps, values approaching 1 indicate regions where incoherent dynamics dominate the total field, typically observed far from generation sites.

**Fig. 6.**
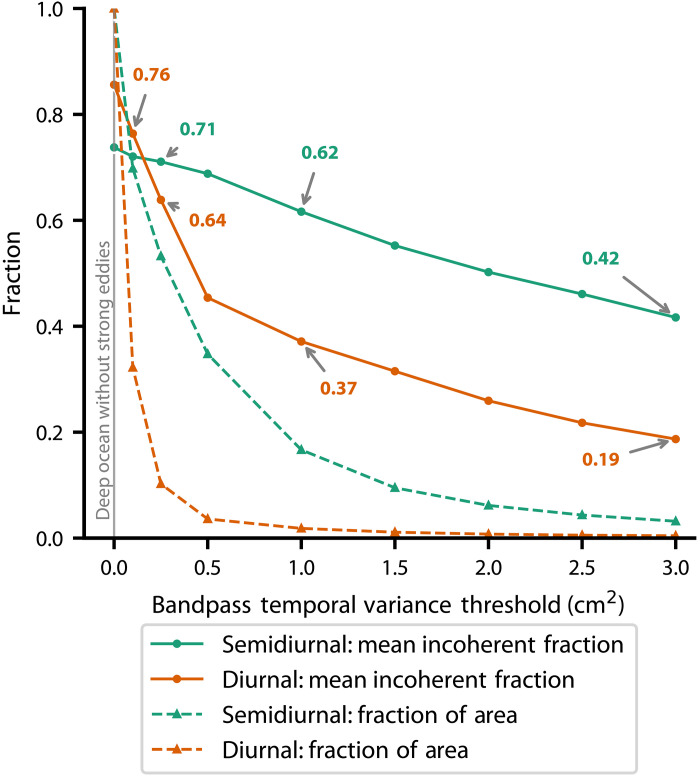
Persistence of non–phase-locked internal tides across energy thresholds in the global ocean. Solid lines show the global mean fraction of internal tide energy that is non–phase locked (incoherent) for semidiurnal (teal) and diurnal (orange) frequency bands, plotted as a function of increasing temporal variance threshold, progressively isolating the most energetic regions. Dashed lines represent the fraction of area exceeding each threshold value. Results reveal that even when only the strongest internal tide regions are considered, a large portion of the internal tide energy remains non–phase locked, indicating the prevalence and persistence of temporally modulated internal tides throughout the world ocean.

This non–phase-locked variability is not a minor perturbation; it is often the dominant component of the total internal tide field (Figs. 5 and 6 and fig. S1). In vast areas of the open ocean, particularly in the far-field of generation sites, the non–phase-locked fraction exceeds 50% of the total internal tide energy, reaching over 70% in the semidiurnal band (Fig. 6 and fig. S1, A to F). This spatial pattern supports the physical mechanism of decorrelation: As the internal tide propagates thousands of kilometers, it continuously interacts with the turbulent mesoscale eddy field, which scatters its energy and breaks its phase relationship with the astronomical forcing. The absolute variance of this signal peaks in the climatically critical Indo-Pacific warm pool, where non–phase-locked internal tides represent a major reservoir of oceanic energy (Fig. 5).

To determine whether this incoherence persists in the most energetic regimes, we analyzed the non–phase-locked fraction as a function of the total internal tide variance (Fig. 6 and fig. S1). This analysis shows that non–phase-locked variability is a fundamental feature of the internal tide field at all energy levels, confirming that incoherence is a dominant component even at major internal tide generation hotspots. Even when restricting the analysis to only the most energetic locations (those in the top few percentiles of internal tide variance), the non–phase-locked component still accounts for a substantial portion of the signal. At the highest energy thresholds, the incoherent fraction remains at 42% for the semidiurnal band and 19% for the diurnal band. These differences are rooted in spatial scales; semidiurnal wavelengths match those of the ocean eddies, allowing for much stronger interaction with the turbulent ocean compared to longer diurnal waves (*40*, *41*). The resulting scale alignment causes semidiurnal waves to scatter readily, breaking down their phase and driving a higher incoherent fraction. Despite these band-specific differences, this sustained variance establishes incoherence as a pervasive and fundamental aspect of internal tide dynamics.

These results are broadly consistent with, and extend, prior global estimates for semidiurnal tides. Previous studies using nadir altimetric wave number spectra suggest that roughly 44% of global semidiurnal internal tide variance is incoherent (*42*). Comparable simulations using coarser, non–data-assimilative HYCOM yield fractions ranging from 41 to 49% (*43*, *44*), and Argo float measurements at 1000 dbar indicate even higher incoherent fractions, reaching up to 85% in the deep ocean (*24*).

However, certain dynamically complex regions remain challenging. Regional patterns of model-data disagreement appear in areas including the northern Pacific and the central Atlantic (Fig. 4). These discrepancies, which may result from localized inaccuracies in bathymetry or model physics (*36*), highlight priority areas for future model development.

### Correcting SWOT observations for total internal tides

**Fig. 7.**
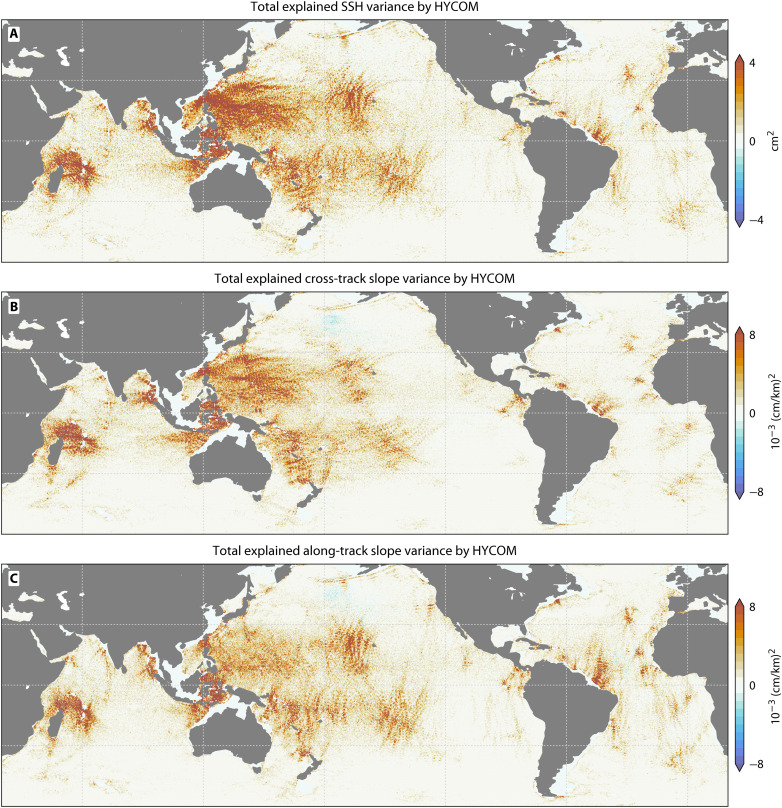
Global maps of HYCOM’s total internal tide predictive skill. Variance explained by HYCOM for (**A**) SSH, (**B**) cross-track slope, and (**C**) along-track slope (on regridded 0.2° bins). These panels evaluate the total internal tide signal (phase-locked plus non–phase-locked) against SWOT altimetry. High explained variance (red) indicates regions where the model successfully captures tidal energy and gradients. Negative values (blue) denote areas of poor model–observation agreement.

Modeling both the phase-locked and non–phase-locked components provides a physically consistent representation of the total internal tide field. This establishes a refined benchmark for satellite altimetry correction. Globally, HYCOM explains a total of 32.57 mm^2^ of variance in SWOT SSH observations, a 59% improvement over the HRET atlas (Fig. 1A). This advantage extends to the sea surface slope, where our framework explains 60% more variance in the cross-track component and 31% more in the along-track component (Fig. 1, B and C). The resulting global map reveals the full extent of the internal tide’s influence, showing interconnected corridors of energy stretching across entire ocean basins (Fig. 7).

**Fig. 8.**
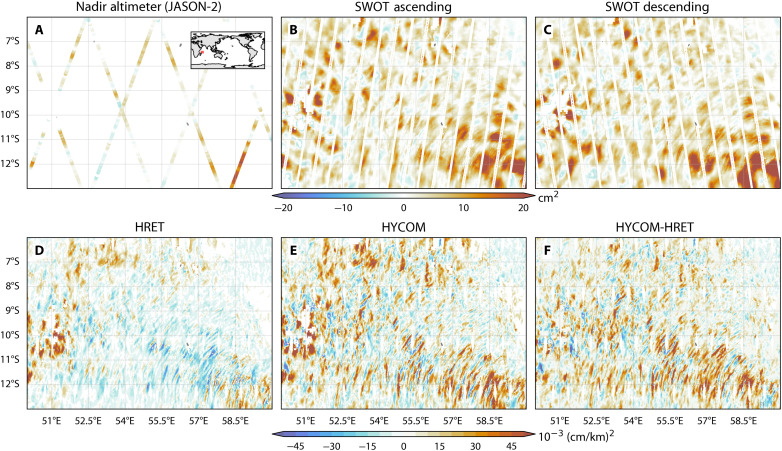
Benchmarking wide-swath resolution and model skill in an internal tide hotspot region. (**A** to **C**) Regional comparison of explained SSH variance by HYCOM in the nadir altimeter JASON-2 (*66*) versus SWOT’s ascending and descending passes. This comparison highlights the resolution gain provided by wide-swath data over traditional nadir observations. (**D** and **E**) Explained cross-track slope variance for the empirical HRET model and HYCOM, respectively. (**F**) The improvement in variance explained by HYCOM relative to HRET. Red values indicate high model skill; in (F), red specifically marks regions where HYCOM outperforms HRET, demonstrating the advantage of physics-based data-assimilative modeling in energetic regimes.

Traditional nadir altimeters provided only sparse, one-dimensional traces (Fig. 8A), while SWOT resolves the full two-dimensional structure of these wave fields (Fig. 8, B and C). Our model’s ability to capture the distinct variance patterns seen by SWOT on both ascending and descending satellite passes confirms that we resolve the directional, small-scale signatures of internal tide propagation. This capability also extends to the sea surface slope (Figs. 7, B and C, and 8, D to F), demonstrating that the simulation accurately reproduces the horizontal structure of the wave field at sub–100-km scales.

Energy from the mesoscale eddy field can potentially leak into our non–phase-locked estimates, as both exhibit variability in overlapping frequency bands. We addressed this potential contamination in two ways. First, we mitigated the most pronounced impacts by masking regions of high eddy variance from our statistics (Fig. 1). Second, we quantified the remaining nontidal noise floor by computing the explained variance in the adjacent subtidal and supertidal frequency bands.

The average explained variance in these nontidal bands is 1.96 mm^2^ (fig. S2), which accounts for only 6% of the total explained tidal variance (32.57 mm^2^). The resulting maps show clear, basin-scale tidal propagation pathways distinct from typical mesoscale structures (Fig. 4). Furthermore, the large separation between the in-band signal and out-of-band noise provides high confidence that our framework resolves physical tidal processes. Quantifying any remaining leakage remains a priority for future work, but these findings represent a first-order, physically consistent validation.

The simulation’s ability to capture a large fraction of the total observed variability validates its physical realism. As noted earlier, this total explained variance does not equal a simple linear sum of the individual components. Nonorthogonality inherent in the harmonic analysis of a time-varying field prevents a strictly linear partitioning of the variance. This limitation, however, does not diminish the model’s overall predictive power.

## DISCUSSION

Resolving the deterministic structure of the full internal tide field considerably reduces a primary source of error in observing mesoscale and submesoscale ocean dynamics from space. While the internal tide remains a complex signal, our validation against SWOT measurements demonstrates that the non–phase-locked component constitutes a globally structured and quantifiable field rather than an unpredictable background noise. By characterizing this observational variance as a predictable feature, this framework provides a robust method to correct high-resolution satellite records.

However, the modeling of coherent and incoherent tides is not yet a fully solved problem. Data from the SWOT calibration and validation (Cal/Val) phase, where high-frequency sampling permitted a direct examination of the aliased M_2_ frequency, reveal that HYCOM still leaves 27% of the total internal tide variance unexplained (*26*). This remaining variance indicates that further model development is necessary to fully capture the internal tide field. Despite this challenge, our framework demonstrates a reliable capability to partition the internal tide signature from the background circulation. Resolving the fine-scale eddies that mediate global heat and carbon transport relies on such separation. The resulting sea surface slope data also facilitate efforts to improve seafloor bathymetry mapping (*32*). Regarding quantitative accuracy targets for SWOT applications, achieving a 59% improvement over the baseline empirical model establishes a refined, physically grounded benchmark. This framework, especially when augmented by emerging dynamic decomposition techniques (*45*–*48*), provides a pathway to further minimize tidal contamination and unlock the full potential of SWOT data. By establishing this baseline, our work advances the mission objectives (*49*) and strengthens our capacity to resolve the physical mechanisms driving Earth’s climate.

These results extend beyond observational correction to enable broader scientific applications, including targeted studies of how the non–phase-locked field directly affects biogeochemical cycles and underwater acoustic propagation (*50*). Advancing beyond the prediction of these discrete tidal signals brings the next major ocean modeling challenge into focus: accounting for the entire internal wave continuum. This study successfully predicts the deterministic, high-energy tidal peaks that dominate this environment. Because these peaks operate within a broader background of stochastic wave energy that remains difficult to simulate, isolating them provides a critical foundation. By extracting the most energetic components of this wave field, our framework establishes the baseline required to guide future modeling efforts targeting the residual continuum energy.

To tackle this residual energy and other remaining challenges, such as refining models in eddy-rich regions and improving fine-scale bathymetric data, advanced structural approaches are required. Embedding the emerging generation of nonhydrostatic models (*51*) as high-resolution regional simulations within global models such as HYCOM provides a promising approach (*52*). This nested configuration allows for the dynamic downscaling of the oceanic energy cascade (*53*). Combining this framework with next generation of in situ platforms will enable a full four-dimensional view of oceanic internal wave dynamics (*54*). Achieving this comprehensive perspective will improve our understanding of current mesoscale processes and how this highly variable internal wave field will evolve on our changing planet.

## MATERIALS AND METHODS

### SWOT satellite SSH observations

We use the SWOT level 3 Ka-band Radar Interferometer (KaRIn) low-rate SSH dataset (alpha v2.0.1), available via the AVISO repository (https://doi.org/10.24400/527896/A01-2023.018). Our primary dataset is the ssha_filtered variable (*55*), which incorporates geophysical corrections including barotropic and baroclinic tides, dynamic atmosphere, wet-troposphere delays, sea-state biases, and time-mean dynamic topography; we subsequently add back the HRET internal tide correction to get the full internal tide signal in the SSH. Furthermore, we removed large-scale ocean signals, including mesoscale eddies by applying the duacs_ssha_karin_2_oi product (*26*, *56*).

Following completion of the calibration and validation fast-sampling phase (1-day repeat orbit, March to July 2023), SWOT transitioned on 21 July 2023 to its nominal 21-day repeat orbit (science phase). On this science orbit, SWOT completes 292 distinct orbits per cycle over ∼21 days, providing near-global coverage between 78°S and 78°N.

During the 21-day repeat cycle, KaRIn acquires SSH across two 60-km swaths on either side of nadir (total 120-km swath width), with a 20-km nadir gap and along- and across-track sampling at 2-km resolution. The revisit frequency varies by latitude—2 repeats per cycle at the equator, rising to ≥6 at high latitudes.

This science-orbit configuration trades reduced temporal revisit (compared to the 1-day Cal/Val phase) for substantially improved spatial sampling and near-global coverage, enabling comprehensive observation of mesoscale and submesoscale ocean structure during the science phase. Analyses in this paper focus exclusively on data from the 21-day science orbit, covering July 2023 to December 2024 and spanning the first 25 cycles.

### Empirical internal tide atlas: HRET

The HRET22 internal tide atlas (merger of versions 8.1 and 14) provides a global, empirically derived estimate of phase-locked internal tide signals based on nearly all available exact-repeat nadir altimeter data spanning 1992 to 2021 (*19*, *57*). The dataset incorporates observations from TOPEX/Poseidon, Jason-1/2/3, GEOSAT, ERS-1/2, and Envisat missions.

HRET provides mode-1 and mode-2 internal tide estimates at five major tidal frequencies—M_2_, S_2_, N_2_, K_1_, and O_1_—on a uniform 0.05° by 0.05° (1/20°) global grid. The final product includes a quality control mask that excludes regions where signal extraction is unreliable because of low signal-to-noise ratio or aliasing effects, effectively identifying areas where internal tide amplitudes are deemed excessively noisy or inconsistent.

### HYCOM forecast ocean model simulations

We use a global, data-assimilative configuration of the HYCOM spanning July 2023 to December 2024. This simulation was executed in two consecutive phases: EXPT 22.3 (July 2023 to August 2024) and EXPT 3.1 (September to December 2024), both sharing identical model setups and data assimilation systems. The model is run on a 1/25° tripolar grid with nominal horizontal resolution of ~3 km at mid-latitudes and 41 hybrid vertical layers. The vertical coordinate system transitions smoothly between *z*-level (depth-based), isopycnal (density-based), and sigma (terrain-following) coordinates, adapting dynamically to local oceanographic conditions (*58*, *59*).

Data assimilation is conducted using the Navy Coupled Ocean Data Assimilation system (*60*), a three-dimensional variational scheme with a 24-hour assimilation window and 3-hour incremental analysis updates. All active nadir altimeter data are assimilated via synthetic profiles to map the geostrophic flow, with the important exception of wide-swath SWOT, which is not included in the data assimilation process. To ensure the model reflects nontidal dynamics, these assimilated nadir observations are detided using state-of-the-art correction models for barotropic (*61*) and internal tides (*19*). Consequently, HYCOM generates the internal tide field from its own internal dynamics rather than from the assimilation of tidal signals in nadir altimeter fields. Assimilated observations also include satellite and in situ sea surface temperature and temperature and salinity profiles from Argo floats, moored buoys, and expendable bathythermographs. Daily mean fields are filtered to exclude tidal variability, enabling improved representation of mesoscale and seasonal ocean dynamics. Because SWOT data are not assimilated, our comparison with SWOT SSH provides an independent assessment: The model has no access to the high-resolution mesocale information exclusively observed by SWOT.

Astronomical tidal forcing is applied using eight major constituents: M_2_, S_2_, N_2_, K_2_, K_1_, O_1_, P_1_, and Q_1_. Barotropic tide accuracy is enhanced through two mechanisms: a topographic wave drag parameterization (*62*) that accounts for unresolved internal wave generation over steep topography and an Augmented State Ensemble Kalman Filter that refines the barotropic tidal signal by estimating optimal forcing correction terms to minimize discrepancies with the Topex/Poseidon Cross-Over (TPXO) data-assimilative tidal model (*63*).

Surface atmospheric forcing is provided by the Navy Global Environmental Model (*64*), which offers fields at 0.17° resolution with 60 vertical levels extending up to 19-km altitude. Subgrid-scale vertical mixing is handled using the K-profile parameterization scheme.

To isolate internal tide signals from the model SSH output, we first regrid the native 1/25° HYCOM hourly fields onto a uniform 4-km grid and then apply two-dimensional Gaussian spatial filters to suppress large-scale and barotropic variability. A 300-km cutoff is used to extract semidiurnal signals, while a 500-km cutoff isolates diurnal variability (*25*). To minimize leakage from coastal effects and large-scale barotropic tides, we mask out grid cells shallower than 750 m (semidiurnal) and 1500 m (diurnal). Throughout this study, we define the filtered SSH as the residual obtained by subtracting the Gaussian-smoothed SSH field from the total HYCOM SSH. This filtered SSH isolates internal tide variability and is used as the primary representation of the internal tide signal. The combined 18-month dataset from EXPT 22.3 and EXPT 3.1 fully overlaps with the first 25 cycles of SWOT’s 21-day science orbit, providing a consistent and high-resolution model baseline for internal tide evaluation.

### Estimation of phase-locked and non–phase-locked internal tides

To quantify both the phase-locked and non–phase-locked internal tide signals, we first carried out all computations on a uniform 4-km grid using filtered SSH from HYCOM. The phase-locked internal tide signal, denoted $\eta_{\text{pl}}$, was extracted via harmonic tidal analysis applied to the filtered SSH time series at each grid point. This analysis was performed for eight principal tidal constituents: M_2_, S_2_, N_2_, K_2_, K_1_, O_1_, P_1_, and Q_1_. The resulting decomposition yielded the harmonic constants—amplitude $A_{n}$, frequency $\omega_{n}$, and phase $\phi_{n}$—at each location on the grid, representing the deterministic, phase-locked component of the internal tide field.

Simultaneously, we isolated internal tide variability within the diurnal and semidiurnal frequency bands by applying a fourth-order Butterworth bandpass filter (*65*) to the same SSH dataset. The filter retained spectral energy between 0.8 to 1.2 cycles per day (cpd) for the diurnal band and 1.8 to 2.2 cpd for the semidiurnal band. The bandpass-filtered SSH, denoted $\eta_{\text{BP}}\left(t\right)$, is defined as

$$\eta_{\text{BP}}\left(t\right)=h\left(t\right)\;*\;\eta \left(t\right)$$ (1)

where *h*(*t*) represents the Butterworth transfer function and ∗ denotes convolution in the time domain.

The phase-locked internal tide signal $\eta_{\text{pl}}\left(t\right)$ was then reconstructed on the 4-km grid using the standard tidal synthesis equation

$$\eta_{\text{pl}}\left(t\right)=\sum_{n}A_{n}\text{cos}\left(\omega_{n}t+\phi_{n}\right)$$ (2)

where the summation index n runs over the eight tidal constituents listed above.

The residual non–phase-locked internal tide signal, $\eta_{\text{npl}}\left(t\right)$, was computed by subtracting the phase-locked reconstruction from the bandpass-filtered SSH

$$\eta_{\text{npl}}\left(t\right)=\eta_{\text{BP}}\left(t\right)-\eta_{\text{pl}}\left(t\right)$$ (3)

Once computed, both the phase-locked and non–phase-locked internal tide signals were regridded from the 4-km HYCOM grid onto SWOT’s 21-day ground track coordinates using a nearest-neighbor search based on a *k*-dimensional tree algorithm. This approach efficiently identifies the closest model grid point to each SWOT track location in space, ensuring spatial alignment between the modeled internal tide fields and the SWOT sampling geometry.

A known challenge of bandpass filtering is the potential for leakage from mesoscale eddies, whose energy can leak into the internal tide frequency bands, particularly in dynamically active regions. To directly address this issue, we completely mask out the regions with strong eddies in our non–phase-locked internal tide signal. These regions are defined as any location where the AVISO-derived sea level variance exceeds 200 cm^2^. While this approach targets the most dominant leakage, our final $\eta_{\text{npl}}$ estimates are likely not entirely free of residual eddy signals.

### Sea surface slope

To assess model performance in representing the horizontal structure of internal tides, we computed sea surface slope components from the SSH fields. Slopes were calculated for both SWOT observations and model outputs on the 2-km SWOT ground track using a first-order, centered finite difference scheme. The local grid spacings, *dx* (cross-track) and *dy* (along-track), were calculated at each point using the Haversine formula to account for Earth’s curvature. Here, *x* and *y* correspond to the cross-track and along-track coordinates, respectively, aligned with the satellite swath geometry. This process yielded two orthogonal slope components: the cross-track slope ($\nabla_{x}\text{SSH}$) and the along-track slope ($\nabla_{y}\text{SSH}$), both defined on an interior grid.

### Explained variance statistics

We quantify model performance for both SSH and its slope using “explained variance” (often referred to as variance reduction), defined as the variance in the SWOT signal that is captured by a given model. This approach follows previously established methodologies (*19*, *20*, *25*, *26*). The explained variance is computed as

$$\text{Explained variance}=\text{Var}\left({\text{Signal}}_{\text{SWOT}}\right)-\text{Var}\left({\text{Signal}}_{\text{SWOT}}-{\text{Signal}}_{\text{Model}}\right)$$ (4)

where Var denotes the temporal variance at a given spatial location. The term Signal represents the variable of interest and their phase-locked and non–phase-locked components, which include the total SSH time series, the cross-track slope ($\nabla_{x}\text{SSH}$), and the along-track slope ($\nabla_{y}\text{SSH}$). To ensure a valid calculation when isolating the non–phase-locked component, the phase-locked signal is removed from the ${\text{Signal}}_{\text{SWOT}}$ before computing the explained variance statistics. A higher explained variance in any of these metrics indicates a better match between the model and observations and is therefore indicative of superior model skill. This same statistic is also computed and averaged for the nontidal frequency bands (1.4 to 1.8 and 2.2 to 2.6 cpd) to provide a rough estimate of the noise floor in the semidiurnal tidal band.
