# Supplementary material for: A global internal tide modeling framework for improving satellite observations of fine-scale ocean circulation
Source: Sci Adv. 2026 Jun 3;12(23):eaee1885. doi: 10.1126/sciadv.aee1885 (PMC13232607; doi:10.1126/sciadv.aee1885)
Supplement: Supplementary file 1 — Figs. S1 and S2 [file sciadv.aee1885_sm.pdf]

Science Advances
AAAS

Supplementary Materials for

A global internal tide modeling framework for improving satellite observations of fine-scale ocean circulation

Badarvada Yadidya *et al.*

Corresponding author: Badarvada Yadidya, yadidyabadarvada@gmail.com

Sci. Adv. **12**, eaee1885 (2026)
DOI: 10.1126/sciadv.aee1885

This PDF file includes:

Figs. S1 and S2

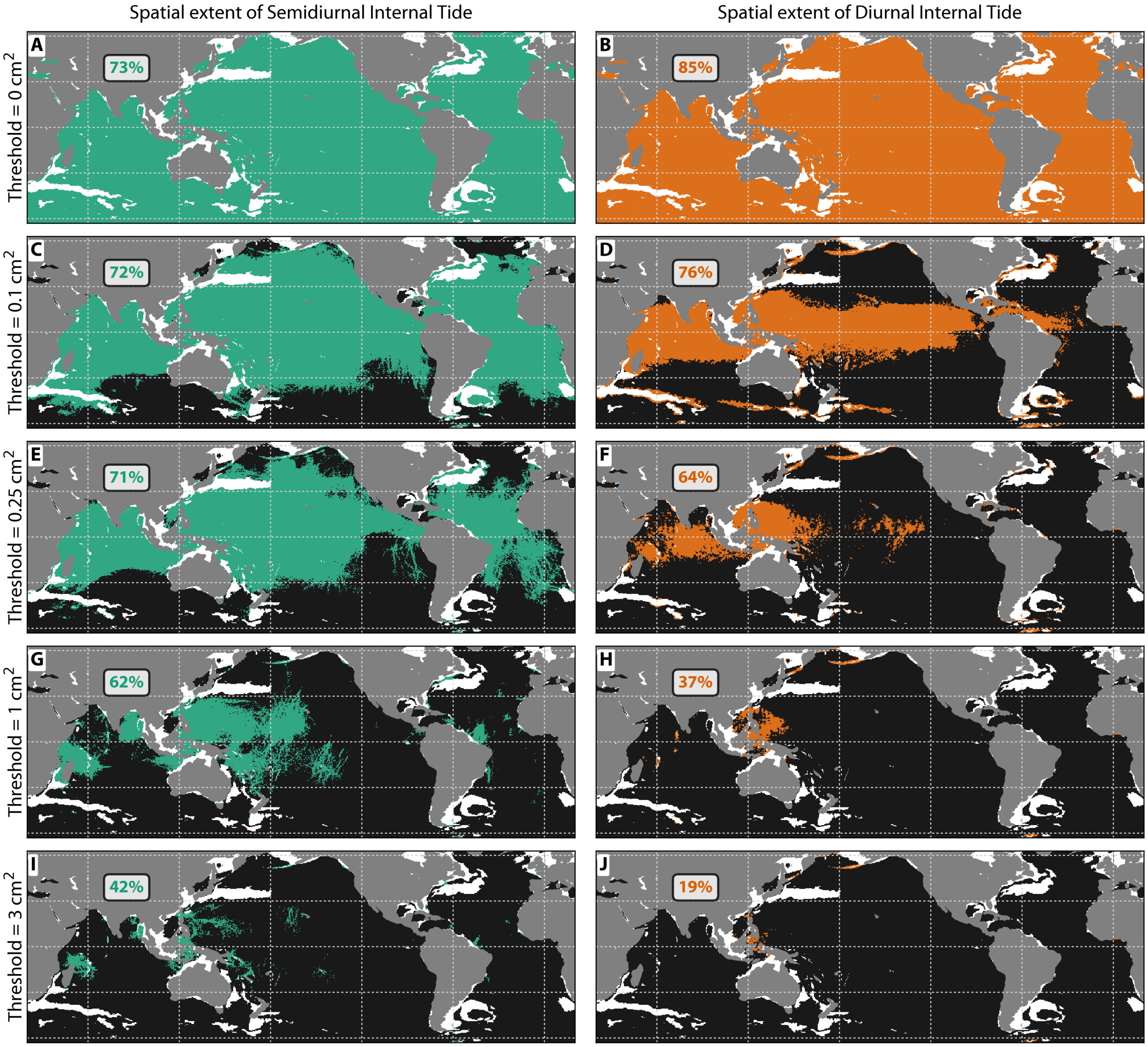

Figure S1: **The global footprint of non-phase-locked internal tides.** Global maps showing the ocean regions where total internal tide variance exceeds specific thresholds, from 0 to 3 cm^2. The analysis is shown for the (**A**, **C**, **E**, **G**, **I**) semidiurnal and (**B**, **D**, **F**, **H**, **J**) diurnal frequency bands. Colored areas meet the variance threshold specified for each row, while black areas fall below it. White regions are masked due to strong mesoscale eddies where the separation of non-phase-locked internal tides is difficult. The percentage in each panel indicates the mean fraction of the total variance contributed by the non-phase-locked (incoherent) component, averaged over the colored regions shown.

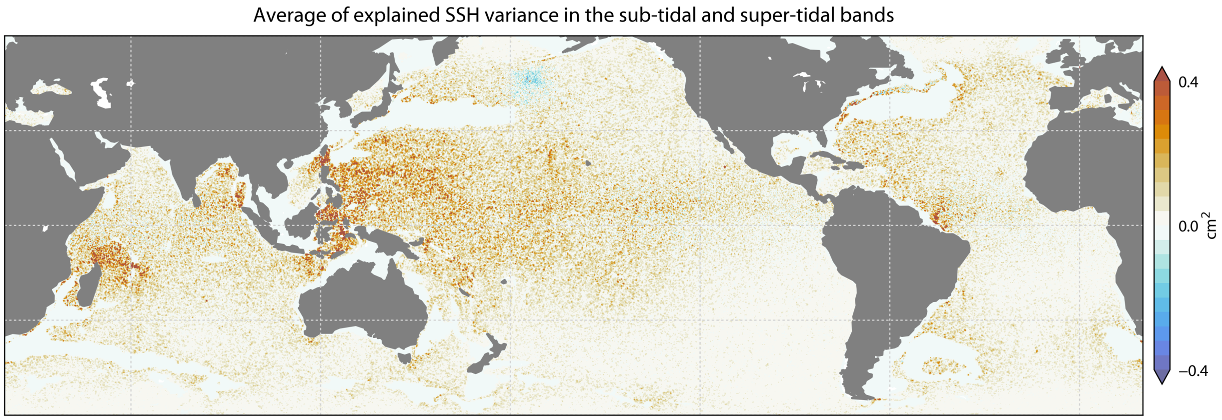

Figure S2: **Estimate of non-tidal variance leakage into the semidiurnal band.** Average explained variance in the subtidal and supertidal frequency bands, representing the noise floor and potential leakage from mesoscale eddies into the semidiurnal internal tide spectrum (data regridded from SWOT tracks onto 0.2° bins).
